# Evaluation of focal liver lesions using triple phase contrast computed tomography among Indian patients 

**DOI:** 10.6026/9732063002001429

**Published:** 2024-10-31

**Authors:** Arjun Mittal, Kumbhar R. R

**Affiliations:** 1 Department of Radiodiagnosis, Krishna Vishwa Vidyapeeth (Deemed to Be University), Karad, Maharashtra, India

**Keywords:** Triphasic computed tomography, detection, characterization, focal liver lesions (FLL), abnormalities, hepatocellular carcinoma (HCC)

## Abstract

Detecting and characterizing focal liver lesions remains a significant challenge in clinical practice. Therefore, it is of interest to evaluate the patients with focal liver lesions using Triphasic computed tomography. Hence, 80 patients spanned for around 18 months to correlate between computed tomography scan findings and final diagnosis. We found male dominancy with high sensitivity for diagnosing hepatocellular carcinoma (HCC) at 73.7%, hemangioma’s at 94.1%, and metastases at 98.4%. Thus, we show that, tri-phasic computed tomography can be widely accepted computed tomography protocol used for assessing liver lesions, allowing for the detection and characterization of most focal liver abnormalities across various pathological scenarios.

## Background:

Research has shown that, among the various liver pathologies (LP), liver masses are particularly significant [1]. Studies have shown that until they are calcified, liver lesions (LL) are not visible on conventional radiographs. As an initial investigation to evaluate liver lesions, U/S is often employed [2]. A study has shown that, focal liver lesions can be either benign or malignant, and their prevalence varies significantly across geographic regions and ethnic groups [3]. These lesions are often benign and may be monitored with examinations in patients without a history of cancer or chronic liver disease (CLD) [4]. In up to 52% of the general population, benign hepatic tumors (BHT) have been reported [5]. Detecting and characterizing focal liver lesions remains a significant challenge in clinical practice. These abnormalities, increasingly identified through diagnostic imaging, require precise differentiation to guide appropriate treatment decisions. Therefore, it is of interest to report and evaluate role of computed tomography in managing focal liver lesions and its contributions to clinical decision-making.

## Materials and Methods:

A prospective observational study was carried out at the KIMS, Karad, Maharashtra, with 80 patients spanning all age groups who were clinically suspected of having focal liver lesions or whose prior imaging had shown non-specific focal hepatic lesions (DFHL). The patients were examined using Triphasic (TP) computed tomography after their personal data, including age and sex, had been recorded. Along with histology, surgical results, ultrasound, and follow-ups, the Triphasic computed tomography examination's findings were compared to the lesions' visibility and enhancement patterns. In order to avoid any potential issues with the contrast medium, patients were urged to refrain from eating or drinking for four hours before the computed tomography scan, according to the imaging protocol. Before the trial, the patient was informed about the risks associated with contrast delivery and their agreement was taken. At first, all patients were placed in supine position. Sections were taken in the hepatic arterial phase (HAP) for 40 seconds, the portal venous phase (PVP) for 60 seconds and delayed phases for 3 to 5 minutes. The pictures were reconstructed at a resolution of 2.5 mm as shown in (Figure 1 see PDF & Figure 2 see PDF).

## Exclusion criteria:

[1] Pregnant women

[2] Those who were contraindicated for CT (*i.e.* hemo-dynamically unstable patient, allergic to contrast media and deranged renal function *etc.,*)

## Statistical analysis:

Diagnostic statistics was assessed for sensitivity, specificity, positive predictive value (PPV), negative predictive value (NPV) and accuracy.

## Results:

Table 1 show that, majority of the patients were from 60-69 years of age (31.25%). Table 2 shows male dominancy with 49 patients in number (61.25%) which was followed by female patients with 31 in number (38.75%) respectively. Table 3 shows that, out of 299 patients 176 showed in HP-L (58.90%) while the remaining 123 showed in HY-L (41.10%) respectively. (Figure 1 see PDF) shows distribution of benign and malignant lesion. (Figure 2 see PDF) shows distribution of HP-L and HY-L respectively. (Figure 3 see PDF) shows distribution of malignant and benign tumor. (Figure 4 see PDF) shows distribution of HY-L among 3 groups *i.e.*, PLN, HAP and PVP. Table 4 shows that maximum number of malignant lesion was seen in 18 patients (100%) with metastases, followed by A (variegated)AA (capsule) in 14 patients (100%) with intrahepatic CCA, then hyper A/A in 5 patients (24%) with HCC and finally, hyper(incomplete)/A/A in 2 patients (100%) with intrahepatic CCA respectively whereas for benign lesion A(puddles)/A/A showed maximum cases with 64 in number (100%) for hemangiomas, followed by A/A/A/ (cleft) with 4 patients (100%) for FNH respectively. Table 5 shows that abscess, adenoma, cyst, HCC, hemangioma , FNH , intrahepatic CCA and metastases all showed statistically significant difference in co-relation of computed tomography and final diagnosis as the p value was <0.001 and <0.003 respectively. (Figure 5 see PDF) shows distribution of HP-L among PLN, HAP and PVP respectively.

## Discussion: 

Although the liver receives 80% of its blood supply from the portal vein and 20% from the HA, primary and secondary neoplastic LL derive their blood supply from the hepatic artery. In the hepatic arterial phase (HAP), HY-L is easily identifiable against the minimally enhancing liver parenchyma (LP). During the PVP, most HL appears as HP-L, contrasting with the strongly enhancing normal liver parenchyma. The conspicuity of a lesion during HAP or PVP depends on its vascularity. In our study, out of the total 299 focal liver lesions seen in 80 patients there were 176 HP-L and 123 HY-L accounting for 59% and 41% of the total (n=299) lesions respectively. On the PVP a greater number of HP-L was identified with greater lesion conspicuity than on other phases especially when lesion was less than 3cm in size. No statistically significant difference was seen between PVP and HAP when size were >3cm. In addition to this, we identified a greater number of hyper-vascular lesions during the HAP compared to PVP and unenhanced phase (UE-P), particularly for lesions smaller than 3 cm. The UE-P scans demonstrated lower sensitivity in detecting small lesions due to the difficulty in distinguishing them from UE-P vessels and biliary dilation. Larger lesions were visible across all phases, with most differences observed in lesions smaller than 3 cm. Triphasic computed tomography enhancement patterns showed 100% sensitivity and specificity for the identification of abscesses, cysts, FNH and intrahepatic CCA. However, sensitivity varied for HCC (73.7%), HMG (94.1%) and Metastases (98.4%), with 100% specificity observed for typical enhancement patterns of each lesion type.

Our findings align with the study by Miller *et al.* found that a larger number of lesions were detected on the HAP than on other phases for lesions smaller than 2 cm and conspicuity of these lesions was higher on the HAP, with significant statistical differences observed between PVP and HAP, PVP and UE-P, HAP and UE-P for lesions smaller than 3 cm. In our study, we grouped lesion sizes as <1 cm, 1-3 cm and >3 cm, while Miller *et al.* categorized them as <1 cm, 1-2 cm, 2-3 cm and >3 cm [6]. Our study was also correlated well with the study done by van Leeuwen *et al.* they identified 11 enhancement patterns, with 6 of these consistently associated with benign conditions and 3 consistently associated with malignant conditions and the other 2 patterns were due metastases and HMG [7].

## Conclusion:

Triphasic computed tomography can be a widely accepted computed tomography (CT) protocol used for assessing liver lesions, allowing for the detection and characterization of most focal liver abnormalities across various pathological scenarios. CT remains pivotal in diagnosing liver diseases. Its widespread use is largely attributed to its ability to provide clear visualization of the liver's anatomical relationships.

## Figures and Tables

**Table 1:** Age distribution

**AGE (years)**	**FREQUENCY**	**PERCENT (%)**
0-9 years	0	0
10-19 years	0	0
20-29 years	3	3.75
30-39 years	6	7.5
40-49 years	17	21.25
50-59 years	26	32.5
60-69 years	25	31.25
70-79 years	3	3.75
80-89 years	0	0
TOTAL	80	100

**Table 2:** Gender distribution

**Gender**	**Number**	**Percent**
Male	49	61.25
Female	31	38.75
Total	80	100

**Table 3:** Distribution of HP-L & HY-L

**Group**	**Number**	**Percentage**
Hypo vascular Lesions(HP-L)	176	58.90%
Hyper vascular Lesions(HY-L)	123	41.10%
Total	299	100%

**Table 4:** Correlation with final diagnosis & HY-L

**Enhancement patterns**	**Malignant lesions**			**Benign lesions**		
	**No**	**%**	**Final Diagnosis**	**No**	**%**	**Final Diagnosis**
A(puddles)/A/A (n=64)	0			64	100	Hemangiomas
A/A/A(cleft) (n=4)	0			4	100	FNH
A(variegated)/A/A(capsule)(n=14)	14	100	HCC	0		
hyper(incomplete)/A/A (n=2)	2	100	Intrahepatic CCA	0		
mixed/mixed/mixed (n=18)	18	100	Metastases	0		
hyper/A/A (n=21)	5	24	HCC			
	15	72	Metastases			
				1	4.5	Adenoma

**Table 5:** Correlation of CT & final diagnosis

**Diagnosis**	**Sensitivity**	**Specificity**	**PPV**	**NPV**	**Accuracy**	**p value**
Abscesses	100	100	100	100	100	< 0.001
Adenoma	0	100	0	99.6	99.6	< 0.003
Cysts	100	100	100	100	100	< 0.001
HCC	73.7	100	100	98.2	98.3	< 0.001
Hemangioma	94.1	100	100	98.3	98.6	< 0.001
FNH	100	100	100	100	100	< 0.001
Intrahepatic CCA	100	100	100	100	100	< 0.001
Metastases	98.4	100	100	98.9	99.3	< 0.001

## References

[1] Venkatesh M (2021). International Journal of Contemporary Medicine Surgery and Radiology..

[2] Rani S, Tripathi P (2018). International Journal of Scientific Study..

[3] Méndez-Sánchez N (2005). Annals of Hepatology..

[4] Schwartz LH (1999). Radiology..

[5] Karhunen PJ (1986). Journal of clinical pathology..

[6] Miller FH (1998). AJR American journal of roentgenology..

[7] van Leeuwen MS (1996). Radiology..

